# The Attitude of Great Writers towards Disease

**Published:** 1892-06-04

**Authors:** 


### The Attitude of Great Writers Towards Disease.

III.—FRENCH SATIRISTS AND THE DOCTORS.

The doctors had a hard time of it in French literature during the sixteenth and seventeenth centuries. It is remarkable to find a company of really worthy men, belonging moreover, to a rich and learned profession, the butts of a satire absolutely unrestrained in its exuberance, extending over so lengthened a period, and exercised by men so different in character and calling as Montaigne, Boileau, Molière, and Le Sage. And it must be taken into account that against the lives of these professors of medicine no very grave accusations are levelled. Even by their enemies' showing they appear to have lived for the most part innocently and laboriously; their learning was undoubted, if not of a very useful description, and they held a high position in the country. La Bruyère, writing at the same time as Molière, reserves all his satire for the quacks, and comments on the fact that the doctors thrive in the teeth of all their satirists. He informs us that though the amount of the fee is settled by the patient, and often consists of gratitude alone, yet these voluntary offerings suffice to enrich them; they marry off their daughters with large portions, and place their sons out in the world to the greatest advantage. There is nothing in all this which speaks the fool or impostor. Where then does the root of the prejudice lie? The answer is best sought in the satires themselves, and Montaigne, who might be said to have started the chorus, is on the whole the writer who throws the most light on the subject. He is anxious to prove to himself and others that his prejudices are not purely hereditary, and therefore unreasonable. His father, grandfather, and great-grandfather died at a ripe age undoctored. Of all his relations, one uncle only, the Sieur de Bussaguet, allowed the doctors to meddle with him, for being Councillor in the Parliament Courts his judgment was unhappily warped by his commerce with other arts. He alone, though the strongest of the whole family, died young. Such examples and his own experience of perfect health till the age of 49 have certainly inclined him against physic, but other considerations are not wanting. In the first place the uncertainty of the whole matter disgusts him. The doctors of each age, he finds, confound their predecessors; each individual contradicts and despises his fellow. In a tone of good-natured irony he rallies his submissive friends on this point. "If your doctor forbids your sleeping, or drinking wine or eating meat, make yourself easy, I will find you another who shall order you all three." The same trait, less comfortably illustrated, is seized by Molière in "L'Amour Medecin": "If your daughter is not bled instantly she is a dead woman." "If you allow her to be bled she cannot survive a quarter of an hour." Montaigne has no notion of submitting to authority himself: "To suffer from colic and be deprived of oysters—here are two evils for one." For he sees no certainty anywhere. How, for instance, he reasons, can each separate ingredient in a mixture get itself disentangled and find its way to the part of the body for which it is intended? And the ideas he has received of experimental physic certainly justify a good deal of scepticism. This is the way they affect to go to work: the physician gazes round forsooth till his fancy is taken by the horn of an elk, let us say; this is to be his remedy. Now he must choose the disease it is to heal, and chance again leads him to the right one. To this must be added, also by guesswork, the right part of the body to be treated, and there remain still to settle at hap-hazard the right conjunction of the planets and the proper season of the year, before diagnosis and remedy are complete. Who will hazard his health on these terms?

But Montaigne has a stronger point in reserve. It is not only the uncertainty and the contradictions of the science, it is its dishonesty he reprobates. For what other word can be applied to that affectation of mystery, that assumption of superior knowledge which can yet condescend to ally itself with the most childish remedies? What can he think of a man who proposes to cure his ailments with the left foot of a tortoise, the dung of an elephant, the liver of a mole, or blood drawn from the wing of a white dove? He cites one example which he was persuaded to try himself as a specific against stone. The cure was to consist of the blood of a goat chosen out of the pasture in the height of summer, and carefully nourished for some weeks on white wine and laxative herbs. All this was duly done, and at the proper season the beast was slain. But with an odd mixture of triumph and disappointment, Montague relates that the goat was found after a post-mortem examination by the cook, to be suffering from precisely the same disease it was intended to cure. Those were not days of homœopathy, and the conclusion was irresistible.

Still more frivolous than the remedies, Montaigne finds the excuses of the doctor when his patient fails to recover: "He has uncovered his arm, has heard the noise of a coach, has half opened the window, or lain on his left side." They must never be blamed, and, moreover, to every happy accident which effects a cure they must lay claim.

All these indictments justify, no doubt, without anything more serious, a considerable degree of dislike and suspicion. Add that most of the physicians are of foreign extraction (*Latineurs*) and take into account the irritation of "*ceste grimace rebarbative et prudente*" and the position of the satirists becomes still clearer.

No doubt the large number of mere charlatans added to the feeling against mediciners, for there is abundant evidence to show how brutal and disgusting many of these quack remedies were, which professed to cure all diseases. No treatment, however, seems to have been more cordially and generally derided than anything of the nature of a water cure. The author of "Gil Blas" thinks bleeding may be overdone; he has no doubt whatever that water drinking is a distinct abomination, leading direct to the grave. Time brings its revenges. If Dr. Sangrado could have taken a glance into the future, he might have been comforted to see the number of those who, little dreaming they are his disciples, now pin their faith to sipping the beverage ("hot, but not boiled"), on the virtues of which he staked and lost his reputation.

The revulsion from these rash empirics produced in the regular and learned members of the profession a habit of mind equally distasteful to the public, and brilliantly distorted in the pages of Molière. Here authority is everything; nothing must be hazarded or undertaken in a hurry. "Better to die according to rules than recover in opposition to them." All this, with much of the swelling self-importance and oracular decisions of the craft, passes gaily before the scene, illustrating the petty absurdities, the peculiarities of garb and manner, which far more than any irregularity of life served, as we have seen, to give a handle for satire. Here are the King's physicians in person, mimicked in every tone and gesture, and fitted with discreetly abusive names in Greek, which Boileau has lent his learning to fabricate. An unreal fantastic assembly at best appears in these "medical" plays of Molière's, with their dancing surgeons and singing apothecaries, more calculated to raise inextinguishable laughter in their victims than bitter retorts. Perhaps the worst indictment after all against the doctors, lies in the attitude they adopted towards their satirist. That attitude of hostility helps us, at any rate, to understand how the joke could be kept up from century to century, only dying out to reappear in fresh form—perhaps not altogether wanting, even in the literature of to-day.

